# AGE-BSA down-regulates endothelial connexin43 gap junctions

**DOI:** 10.1186/1471-2121-12-19

**Published:** 2011-05-16

**Authors:** Chi-Young Wang, Hung-Jen Liu, Heng-Ju Chen, Yi-Chun Lin, Hsueh-Hsiao Wang, Ta-Chuan Hung, Hung-I Yeh

**Affiliations:** 1 Department of Veterinary Medicine, College of Veterinary Medicine, National Chung Hsing University, 250 Road Kuo Kuang, Taichung 402, TAIWAN; 2 Institute of Molecular Biology, College of Life Sciences, National Chung Hsing University, 250 Road Kuo Kuang, Taichung 402, TAIWAN; 3 Graduate Institute of Biotechnology, National Ping-Tung University of Science and Technology, 1 Road Shuefu, Pingtung 912, TAIWAN; 4 Departments of Internal Medicine and Medical Research, Mackay Memorial Hospital, 45 Road Minsheng, New Taipei City 251, TAIWAN; 5 Department of Nursing, Mackay Medicine, Nursing and Management College, 92 Road Shengjing, Taipei 112, TAIWAN; 6 Department of Medicine, Mackay Medical College, 46 Road Zhongzheng, New Taipei City 252, TAIWAN

## Abstract

**Background:**

Advanced glycation end products generated in the circulation of diabetic patients were reported to affect the function of vascular wall. We examined the effects of advanced glycation end products-bovine serum albumin (AGE-BSA) on endothelial connexin43 (Cx43) expression and gap-junction communication.

**Results:**

In human aortic endothelial cells (HAEC) treated with a series concentrations of AGE-BSA (0-500 μg/ml) for 24 and 48 hours, Cx43 transcript and Cx43 protein were reduced in a dose dependent manner. In addition, gap-junction communication was reduced. To clarify the mechanisms underlying the down-regulation, MAPKs pathways in HAEC were examined. Both a MEK1 inhibitor (PD98059) and a p38 MAPK inhibitor (SB203580) significantly reversed the reductions of Cx43 mRNA and protein induced by AGE-BSA. Consistently, phosphorylation of ERK and p38 MAPK was enhanced in response to exposure to AGE-BSA. However, all reversions of down-regulated Cx43 by inhibitors did not restore the functional gap-junction communication.

**Conclusions:**

AGE-BSA down-regulated Cx43 expression in HAEC, mainly through reduced Cx43 transcription, and the process involved activation of ERK and p38 MAPK.

## Background

Diabetes is known to accelerate the process of atherosclerosis, in which endothelial dysfunction plays a key role [1]. Previous studies have shown that diabetes alters the expression of a variety of molecules involved in maintenance of endothelial function [2]. Animal experiments also demonstrated that induction of diabetes, either alone or on top of hyperlipidemia, suppress the expression of endothelial gap junctions [3,4]. Gap junctions are cell membrane channels made of paired hexamers of connexins, which allow exchange of ions and small signaling molecules between the cytoplasmic compartments of adjacent cells. In mammals endothelial cells mainly express connexin43 (Cx43), Cx40, and Cx37 [5], of which Cx43 is by far the predominant in the cultured endothelial cells [6,7]. Several reports have shown that endothelial Cx43 gap junctions are down-regulated by factors causing endothelial dysfunction [8], such as aging [9], hypertension [10], and arsenic trioxide [11], the last of which was even reported to induce endothelial lesion. In contrast, other factors causing endothelial dysfunction, such as oscillatory shear stress, were shown to enhance Cx43 expression [12].

One key offending factor underlying the toxic effects of diabetes is glucose, the high level of which had been proved to affect the activities of endothelial cells in many aspects, including suppression of gap junctions [13,14]. Since diabetes is associated with a more severe form of vascular disease, we suspected that molecules underlying the detrimental effects of diabetes other than glucose also affect endothelial gap junctions.

During chronic exposure to elevated blood glucose advanced glycation end products (AGE) are generated in the circulation. AGE are also versatile molecules and have been reported to possess multiple actions in the vascular wall, such as changes of release of cytokines, induction of expression of cell adhesion molecules, impairment of endothelial vasodilatation, and triggering of chronic inflammation [15]. However, the effect of AGE on endothelial gap junctions remained unclear. To this end, we examined the expression of Cx43 as well as the gap-junction communication in human aortic endothelial cells (HAEC) treated with AGE and explore underlying mechanisms. A previous study had shown that the average serum AGE levels in diabetic patients ranged between 28.8 and 87.2 μg/ml and may reach 160-500 μg/ml in severe cases [16].

## Methods

### Generation of AGE-BSA

AGE-BSA was prepared by incubation of BSA (Fraction V, Sigma Chemical Co., St. Louis, MO, USA) at a concentration of 50 mg/ml with 0.5 M glucose in 10 mM phosphate-buffered saline (PBS) containing 0.5 mM EDTA, pH 7.4, at 37°C for 12 weeks. For a control group, the same concentration of BSA was incubated with PBS containing EDTA without glucose for 12 weeks. Free glucose was removed by extensive dialysis against PBS. The brown color of AGE-BSA showed the typical appearance of AGE. SDS-PAGE analysis showed the formation of AGE-BSA monomer and dimer with molecular weights approximately equaling to 78 and 157 kDa, respectively. However, unglycated BSA was about 69 kDa. The ratio of relative fluorescence intensities of AGE-BSA to unglycated BSA was approximately 64 folds in our preparations. Results were consistent with other's report [17]. For a control group, BSA was processed as above procedures. BSA and AGE-BSA were stored at -70°C until use.

### Cell culture

HAECs (Cascade Biologics) were seeded in 1% gelatin-coated plasticware and incubated at 37°C under a humidified 95% air and 5% CO_2 _atmosphere. Cells grown to confluence were dissociated with 3 ml of 0.25% trypsin-EDTA (GIBCO) at 37°C for 3 min. The suspension was diluted with 7 ml medium 200 supplemented with LSGS, centrifuged at 1200 rpm for 8 min, and resuspended in the culture medium. Cells were then replated in 35-mm Petri dishes (5 × 10^4 ^cells per cm^2^) and allowed to grow to confluence or seeded at the same density onto 12-mm glass coverslips coated with 1% gelatin (Sigma). Cells of passage 4 to 6 were used in the subsequent experiments. To evaluate the relative mRNA level of Cx37, Cx40, and Cx43, human umbilical vein endothelial cells (HUVEC, from PromoCell), maintained in endothelial cell growth medium (EGM, from PromoCell) were used as a reference. The confluent cells were treated with AGE-BSA at series concentrations of 25, 50, 100, 250, and 500 μg/ml for 24 and 48 hours. For a control group, cells were treated with BSA at series concentrations of 25, 50, 100, 250, and 500 μg/ml for 24 hours. For examination of activation of MAPK signaling pathways, cells were incubated with AGE-BSA at concentrations of 25, 50, 100, 250, and 500 μg/ml for 2 and 6 hours. For time course experiments, cells were incubated with AGE-BSA at 500 μg/ml for 0, 0.5, 1, 2, 4, and 6 hours. For drug treatments, cells were treated with 40 μM of PD98059 (Sigma), 15 μM of SB203580 (Sigma), and 10 μM of SP600125 (Sigma) for 30 min and then incubated with 500 μg/ml AGE-BSA for 24 hours.

### Western blotting

Cells were collected in RIPA buffer containing 150 mM NaCl, 5 mM EDTA, 1% NP40, 2 mM PMSF, and 50 mM Tris-HCl, pH 7.4 or SB buffer containing 20% SDS, 0.1 M Tris-HCl, pH 6.8, and 10 mM EDTA followed by sonication for 30 sec. Thirty microgram of sample was loaded in each lane, resolved by 12% SDS-PAGE, and transferred onto a PVDF membrane (Amersham, UK). The membrane was incubated with the monoclonal anti-Cx43 antibody (1:1000; Chemicon), anti-Cx43 antibody (1:1000; BD Biosciences), anti-β actin antibody (1:2000; Chemicon), anti-ERK antibody (1:1000; Cell signaling), anti-p38 MAPK antibody (1:1000; Biosource), anti-JNK antibody (1:2000; Cell signaling), anti-phosphorylated ERK antibody (1:1000; Cell signaling), anti-phosphorylated p38 MAPK antibody (1:1000; Biosource), and anti-phosphorylated JNK antibody (1:1000; Cell signaling) at room temperature for 1 hour. After three washes with TBST (20 mM Tris pH 7.6, 150 mM NaCl, 0.1% Tween 20), a horseradish peroxidase-conjugated mouse anti-rabbit or donkey anti-mouse IgG (1:3000 in TBST plus 10% BSA) was added, and an enzyme-linked chemiluminescence system (ECL; Amersham, UK) was applied to check the bound antibody.

### Real-time PCR and Semi-quantitative RT-PCR

Total RNA was extracted using Trizol reagent (Invitrogen Life Technologies, Carlsbad, USA) according to the manufacturer's instructions. After phenol treatment and drying, RNA was dissolved in RNase-free water for determination of concentration using spectrophotometer. RNA quality was checked on agarose electrophoresis. For real-time PCR analysis, endothelial cDNA were amplified with primers specific for Cx43 (sense: 5'-CCT CTC GCC TAT GTC TCC TC-3') (antisense: 5'-GCT CAC TTG CTT GCT TGT TG-3'), Cx37 (sense: 5'-CGT AGA GCG TCA GAT GGC-3') (antisense: 5'-GCA CAC TGG CGA CAT AGG-3'), Cx40 (sense: 5'-TCA ATC CCT TCA GCA ATA ATA TG-3') (antisense: 5'-GTG ACC TGG TGA GAC TCC-3'), and β-actin (sense: 5'-TCC TGT GGC ATC CAC GAA ACT-3') (antisense: 5'-GAA GCA TTT GCG GTG GAC GAT-3') using iQTM SYBR Green Supermix reagent and detected with iQTM Single Color Real-Time PCR Detector System (all from Bio-Rad, California, USA). Relative mRNA levels were normalized with the corresponding levels of β-actin.

For Semi-quantitative RT-PCR, total RNA (2 μg) was reverse-transcribed into complementary DNA (cDNA) using the SuperScrip™ II reverse transcriptase and random primers (Invitrogen Life Technologies) according to the manufacturer's recommendations and diluted with DDW to a final volume of 20 μl. The optimal number of PCR cycles was determined for Cx43 (Cx-1: 5'-TCTGAGTGCCTGAACTTGC-3' and Cx-2: 5'-ACTGACAGCCACACCTTCC-3') and GADPH (GA-1: 5'-CATTGACCTCAACTACATGG-3' and GA-2: 5'-TTGCCCACAGCCTTGGCAGC-3') so that the amplification process was conducted during the exponential phase of amplification. Amplification of the GADPH gene transcript was used as the internal control to stringently control for any variability in RNA degradation and RT efficiency. Reactions were carried out in a 50 μl final reaction volume.

### Immunofluorescence detection

Confluent cells grown on coverslips were fixed with methanol at -20°C for 5 min. After blocking with 0.5% BSA, the cells were incubated with the anti-Cx43 antibody (1:1000; Chemicon) at 37°C for 1 hour, followed by incubation with a CY3 conjugated donkey anti-mouse antibody (Chemicon). The cells were then incubated with FITC-conjugated lectin Ulex europaeus agglutinin-1 (UEA-1; 10 μg/ml; Sigma) for 1 hour to confirm the identity of endothelial cells [18] or bisbenzamide (1 μg/ml; Sigma) for 15 min. Finally, the cells were mounted and examined using a Leica TCS SP confocal laser scanning microscope.

### Scrape loading assay

Gap-junction communication in HAECs treated with 250 and 500 μg/ml of AGE-BSA for 24 and 48 hours was evaluated by scrape loading assay. For drug experiments, HAEC were respectively pretreated with i) 40 μM of PD98059 and ii) 15 μM of SB203580 prior to addition of the AGE-BSA (500 μg/ml) for 24 hours. The medium of confluent cells were removed and rinsed with Hank's balanced salt solution (GIBCO). A 27-gauage needle was used to create multiple scrapes through the cell monolayer in the presence of PBS containing 0.5% rhodamine-dextran and 0.5% Lucifer yellow. After 3 min of incubation at room temperature, the culture was rinsed three times and then incubated for 5 min in medium 200 supplemented with LSGS to allow the loaded dye to transfer to adjoining cells. The cells were viewed and recorded using a fluorescence microscope.

### MTT assay

Cells were incubated in medium 200 supplemented with LSGS containing 0.5 mg/ml MTT [3-(4,5-dimethylthiazol-2-yl)-2,5-diphenyltetrazolium bromide]. After 1 hour, the MTT solution was removed and dimethylsulfoxide (100 μl/well) was added.

Absorbances (550 nm) of the supernatant were read using a microplate spectrophotometer (Spectra Max 340; Molecular Devices, Sunnyvale, CA). Cell viability was expressed as the percentage of the absorbance values of treated cells to controls.

### Analysis

Densitometric scanning and analysis were performed on immunoblots and gel images using Imagemaster (Amersham Pharmacia Biotec, NJ, USA). Within each lane, total amounts of bands of Cx43 were divided by those of actins as loading controls. For RT-PCR, amounts of Cx43 were divided by those of GADPH. For the scrape loading assay, the area between the bilateral edges of lucifer yellow transfer and the scrape line was measured. The value of total amount of each sample was presented as mean (%) ± S.E. The significant differences are analyzed by *t*-test.

## Results

**Figure 1:**
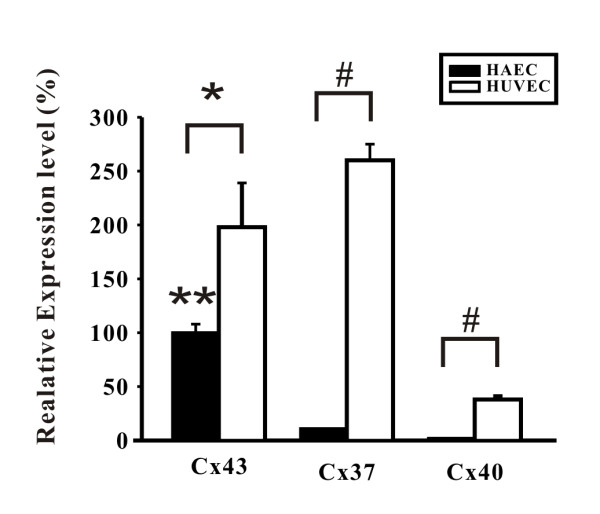
**Analysis of the mRNA profile of Cx37, Cx40, and Cx43 in HAEC and HUVEC showed distinct expression patterns in each type of cells and in HAEC Cx43 was the most abundant transcripts**. Real-time PCR showed the existence of Cx37, Cx40, and Cx43 transcripts. Cx43 and Cx37 were the most abundant connexin transcripts expressed in HAEC and HUVEC, respectively. In HAEC, compared to Cx37 and Cx40, Cx43 was more than 10-fold abundant. *, p < 0.05; #, p < 0.005, HAEC vs. HUVEC. **, p < 0.01 for Cx43 vs. each of Cx37 and Cx40 in HAEC.

**Figure 2:**
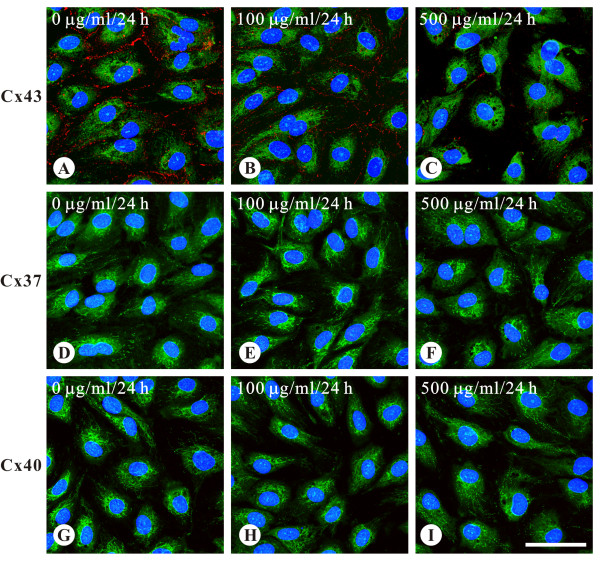
**Expression of Cx37, Cx40, and Cx43 gap junctions in HAEC evaluated by immunoconfocal microscopy showed that in untreated cells Cx43 was abundantly expressed at the cell borders but Cx37 and Cx40 were rarely observed**. AGE-BSA reduced Cx43 signals in a dose-dependent manner. Images were respectively obtained from cells treated with 100 μg/ml and 500 μg/ml of AGE-BSA for 24 hours. Compared with the control group (A), Cx43 gap junctions (red spots) are reduced as the dose of AGE-BSA increases (B and C). Note no conceivable signals of Cx37 and Cx40 were found in all cells. Blue label, nucleus. Green label, lectin UEA1. The concentration of AGE-BSA and the incubation time are indicated in the upper left of each image. h, hours. All images are of the same magnification. Bar, 50 μm.

Real-time PCR showed that Cx37, Cx40, and Cx43 existed in both HAEC and HUVEC. However, the expression levels differed markedly (Figure 1). Regarding HAEC, Cx43 transcripts were more than 10-fold abundant, compared to Cx37 or Cx40 (Cx43 vs. Cx37 or Cx40, both P <0.01), in contrast to HUVEC, in which Cx37 was the most abundant. In addition, the relative mRNA expression levels of individual connexins in HAEC were lower than those in HUVEC (all P <0.05, see Figure 1). Consistently, immunoconfocal examination of the untreated HAEC showed that Cx43 was abundantly expressed at the cell borders, typical for gap junctions, but Cx37 and Cx40 were rarely seen (Figure 2). Treatment with AGE-BSA for 24 hours at doses of 100 and 500 μg/ml lead to a marked reduction of Cx43 and Cx37 and Cx40 remained rarely seen (Figure 2). Therefore, the following experiments in HAEC were focused on Cx43.

**Figure 3:**
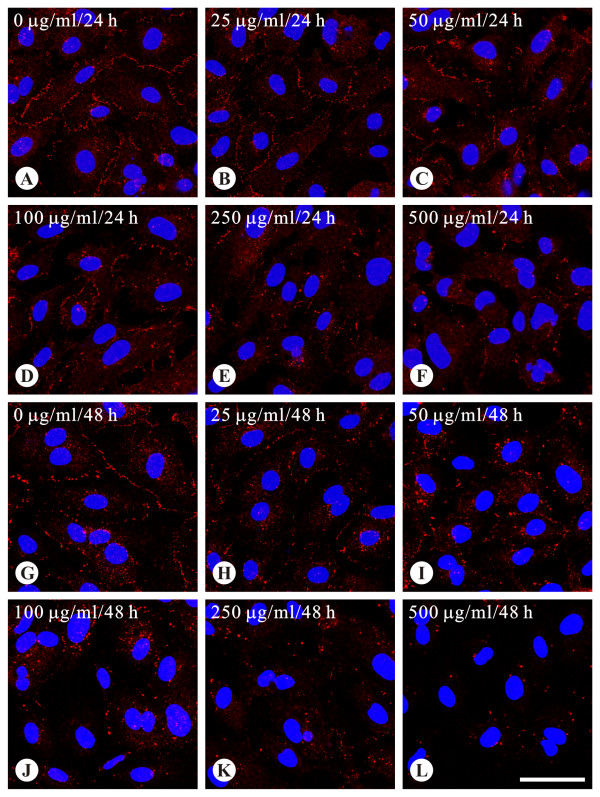
**Reduced expression of endothelial Cx43 gap junctions by AGE-BSA, as evaluated by immunoconfocal microscopy**. The levels of Cx43 decreased as the dose of AGE-BSA increased regardless of 24 or 48-hour treatment. Images A-F and G-L were respectively obtained from cells treated with AGE-BSA for 24 and 48 hours. Compared with the control group (A and G), Cx43 gap junctions (red spots) are reduced as the dose of AGE-BSA increases (B-F and H-L). Blue label, nucleus. The concentration of AGE-BSA and the incubation time are indicated in the upper left of each image. h, hours. All images are of the same magnification. Bar, 50 μm.

**Figure 4:**
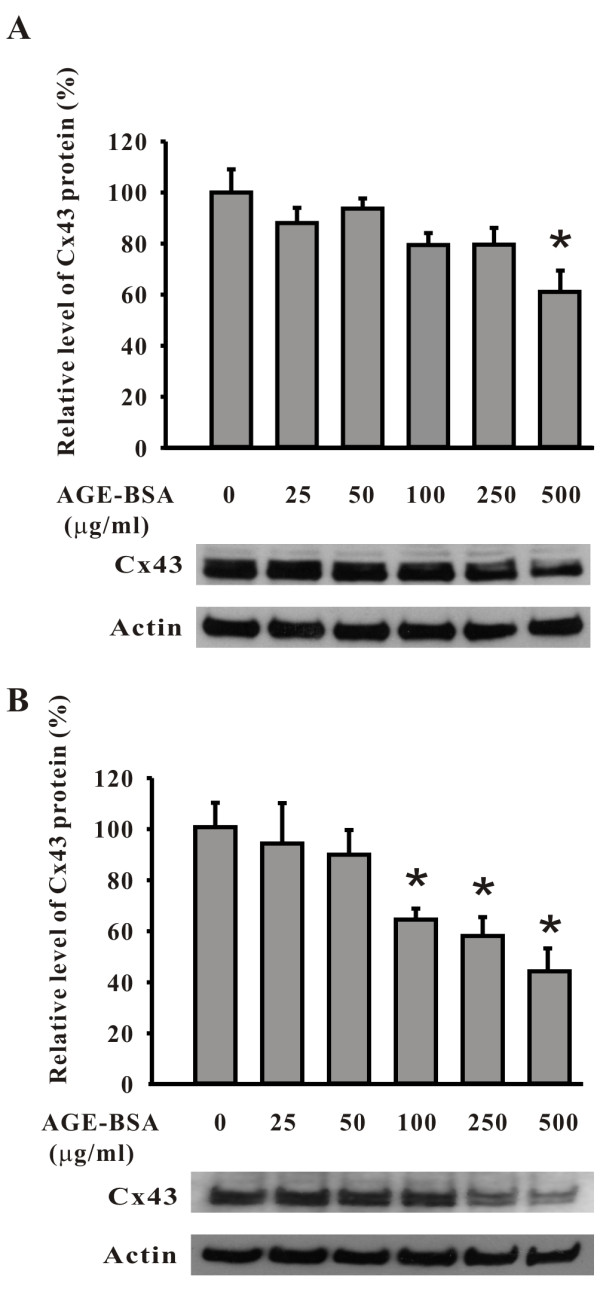
**Dose-dependent reduction of expression of Cx43 protein by AGE-BSA, as detected by Western blotting**. Note that a dose-dependent reduction is seen in cells treated with AGE-BSA for both 24 (A) and 48 hours (B). For each dose of treatment, the relative level of the total amount of Cx43 protein is shown in the histogram at the top of the blot. The relative expression levels of Cx43 protein significantly reduced to 61.2 ± 7.3% and 43.9 ± 8.8% after cells exposure to 500 μg/ml of AGE-BSA for 24 and 48 hours, respectively. *, p < 0.05 compared to the untreated cells.

**Figure 5:**
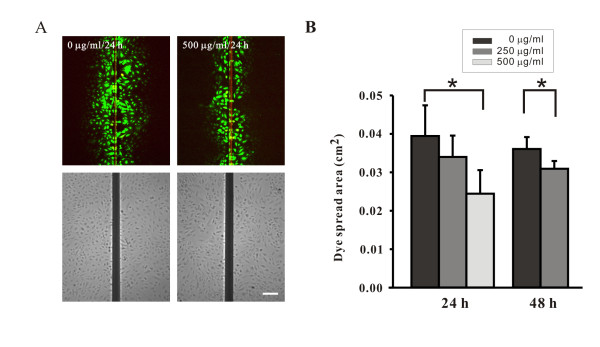
**Reduction of gap-junction communication by AGE-BSA, as evaluated using scrape loading assay**. A. Bottom images are phase-contrast micrographs of the upper fluorescent images. The concentration of AGE-BSA (in μg/ml) is indicated in the upper left of each paired images. B. Analysis of the area between the bilateral edges of lucifer yellow transfer and the scrape line is shown in the histogram. Bar, 150 μm. Note that significant decrements were seen in cells treated with 250 μg/ml for 24 and 48 hours, and 500 μg/ml for 24 hours, respectively. *, p < 0.05 compared to the untreated cells. h, hours. The inhibition of gap-junction communication at 500 μg/ml of AGE-BSA for 48 hours was not quantified because cells were not well contacted.

**Figure 6:**
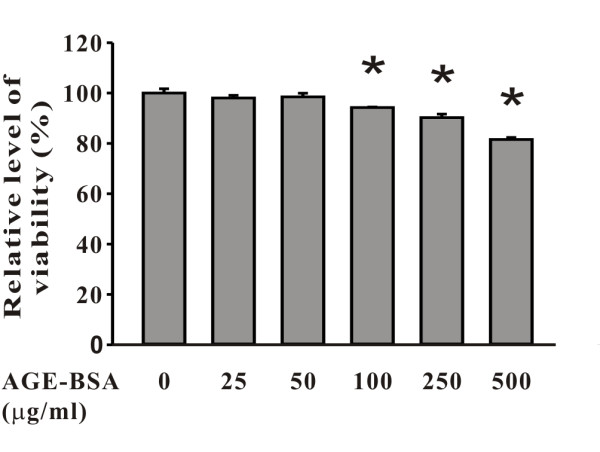
**Dose-dependent reduction in viability of cells treated with AGE-BSA, as detected by MTT assay**. Note a dose-dependent reduction is seen in cells treated with AGE-BSA for 24 hours. For each dose of treatment, the relative level of the total amount of cells is shown in the histogram. The relative viability of cells significantly reduced to 94.2 ± 0.2%, 90.2 ± 1.4%, and 81.6 ± 0.8% post exposure to 100 μg/ml, 250 μg/ml, and 500 μg/ml, respectively. *, p < 0.05 compared to the untreated cells.

In cells treated with AGE-BSA at doses from 25 to 250 μg/ml for 24-48 hours, no changes of the cell density and morphology were observed (Figure 3). However, cells became slightly retracted and reduced in density after treated with 500 μg/ml of AGE-BSA for more than 24 hours (Figure 3L). For Cx43, regardless of the 24 or 48 hours treatment, the levels of expression gradually decreased as the dose of AGE-BSA increased (Figure 3). Western blotting also verified the dose-dependent effect of AGE-BSA on Cx43 expression (Figure 4). After exposure to 500 μg/ml of AGE-BSA for 24 hours (Figure 4A) and 48 hours (Figure 4B), the relative expression levels of Cx43 protein were respectively reduced to 61.2 ± 7.3% and 43.9 ± 8.8% (both P <0.05, compared to the control groups). A control group of cells treated with dialyzed, long term stored BSA for 24 hours showed no changes of Cx43 proteins (Additional file 1 Figure S1). There was no difference in decreasing trends or patterns of Cx43 proteins extracted from AGE-BSA-treated cells with lysis buffers containing NP40 or SDS as well as no difference in Cx43 expression when the samples were detected using anti-Cx43 antibodies from various sources (Additional file 2 Figure 2S and Additional file 3 Figure 3S). The function of gap-junction communication was checked using the method of scrape-loading/dye transfer (Figure 5A). The areas of dye transfer were significantly reduced in cells treated with 500 μg/ml of AGE-BSA for 24 hours and 250 μg/ml of AGE-BSA for 48 hours (Figure 5B; both P <0.05). Moreover, because cells were not well contacted after exposure to 500 μg/ml of AGE-BSA for 48 hours, the inhibition of gap-junction communication was unable to quantify (Figure 5B). The effects of AGE-BSA on viability of cells after exposure for 24 hours were assessed using MTT assay. The relative viability of cells was 94.2 ± 0.2% (100 μg/ml AGE-BSA), 90.2 ± 1.4% (250 μg/ml AGE-BSA), and 81.6 ± 0.8% (500 μg/ml AGE-BSA), respectively (all P <0.05, compared to the control groups) (Figure 6).

**Figure 7:**
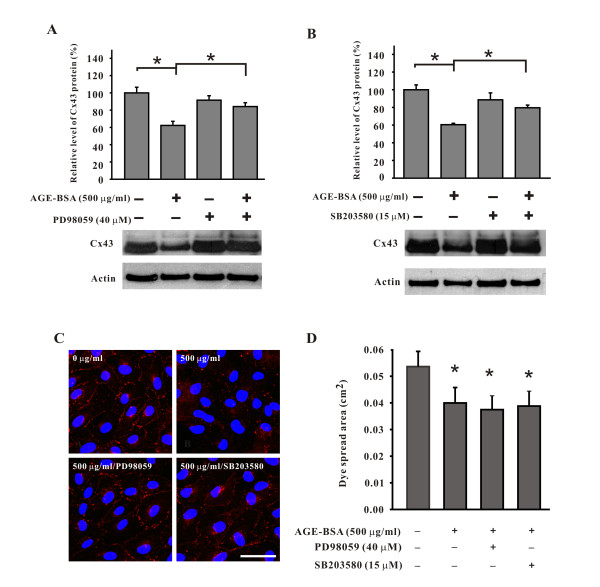
**PD98059 and SB203580 respectively blocked the down-regulating effect of AGE-BSA on Cx43 protein (as detected by Western blotting and immunoconfocal microscopy) but not the effect on the gap-junction communication (by scrape loading)**. Note the relative expression levels of Cx43 in cells treated with AGE-BSA (500 μg/ml; 24 hours) were reversed from 62.3 ± 4.8% to 84.1 ± 4.4% by PD98059 (A) and from 60.5 ± 1.6% to 79.6 ± 2.9% by SB203580 (B), respectively. The results were further confirmed by immunoconfocal microscopy (C). The inhibition of gap-junction communication by AGE-BSA (500 μg/ml; 24 hours) was not rescued in the presence of PD98059 and SB203580 (D). *, p < 0.05 compared to the untreated cells. Images in (C) were obtained from the control group, cells treated with AGE-BSA, cells treated with AGE-BSA and PD98059, and cells treated with AGE-BSA and SB203580, respectively. Note that Cx43 gap junctions (red spots) were reversed near the level of the control group by PD98059 and SB203580. Blue label, nucleus. The concentrations of AGE-BSA and inhibitor used are indicated in the upper left of each image. All images are of the same magnification. Bar, 50 μm.

To explore whether alteration of Cx43 expression by AGE-BSA was regulated through MAPK cascades, three signal pathways, ERK, JNK, and p38 MAPK were separately blocked before addition of AGE-BSA. PD98059, an MEK1 inhibitor which blocks ERK pathway, significantly reversed the reduction of Cx43 proteins in cells treated with AGE-BSA for 24 hours (relative expression levels, without PD98059, 62.3 ± 4.8%; with PD98059, 84.1 ± 4.4%, p < 0.05 see Figure 7A). Similarly, SB203580, a p38 MAPK inhibitor, also significantly reversed the reductions (without SB203580, 60.5 ± 1.6%; with SB203580, 79.6 ± 2.9%, p < 0.05 see Figure 7B). The basal levels of Cx43 proteins were not affected after the addition of these inhibitors. In contrast, no such effects were seen in cells treated with SP600125, a JNK inhibitor (data not shown). Immunoconfocal microscopy also confirmed that AGE-BSA induced reduction of Cx43 was reversed by PD98059 or SB203580 to the levels comparable to the control group and the reversed Cx43 mainly located at cell borders (Figure 7C). The gap-junction communication was measured when cells were treated with 500 μg/ml of AGE-BSA for 24 hours in the presence of either PD98059 (40 μM) or SB203580 (15 μM). Whereas Cx43 proteins returned to approximately the control levels (Figure 7A-C), the areas of dye transfer remained close to those of cells treated with 500 μg/ml of AGE-BSA for 24 hours (all P <0.05, compared to the control groups; see Figure 7D). Taken together, our results indicated the reversed Cx43 by MEK1 or p38 MAPK inhibitor remained at the cell borders but exhibited impaired communication function.

**Figure 8:**
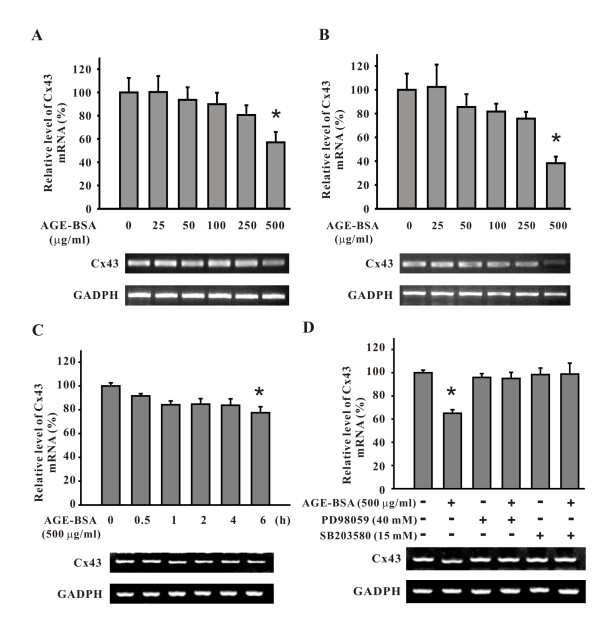
**Reduced transcription of Cx43 by AGE-BSA and partial recovery of reduced Cx43 transcript by each of PD98059 and SB203580, as detected by semi-quantitative RT-PCR**. Note that a dose-dependent reduction is seen in cells treated with AGE-BSA for both 24 (A) and 48 hours (B); a time-dependent reduction is seen in cells treated with 500 μg/ml of AGE-BSA for different time periods (C). The relative transcription levels of Cx43 of AGE-BSA-treated cells were significantly reversed by PD98059 from 65.1 ± 2.1% to 95.1 ± 5.3% or by SB203580 from 65.1 ± 2.1% to 98.8 ± 9.4% (D). For each dose of treatment, the relative level of Cx43 transcripts is shown in the histogram at the top of the blot. h, hours. *, p < 0.05 compared to the untreated cells.

**Figure 9:**
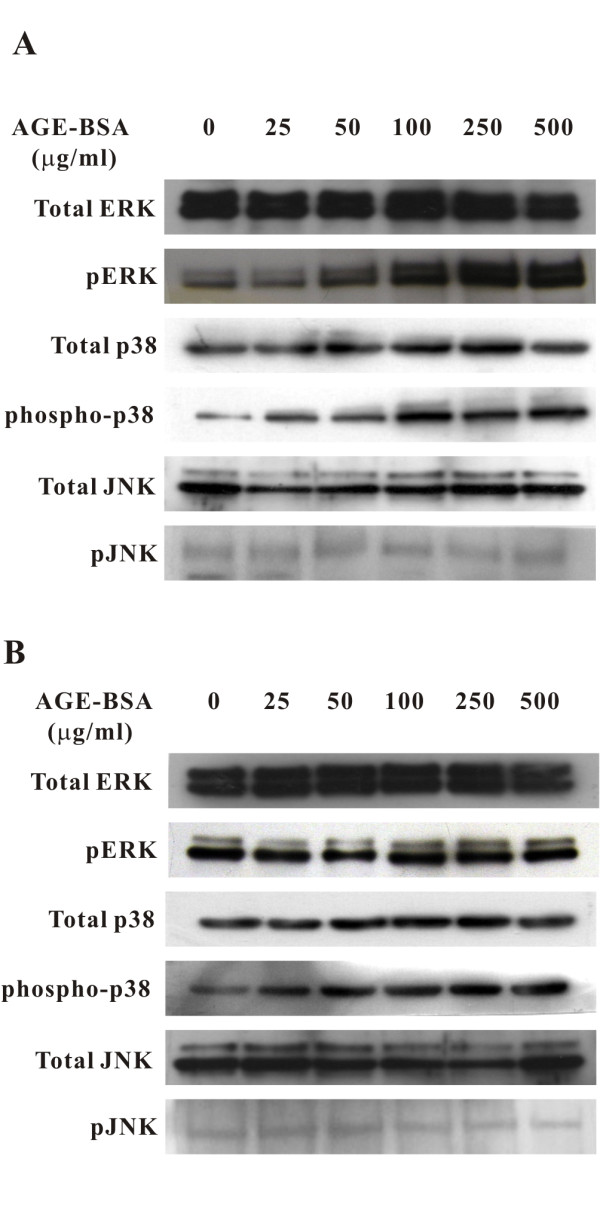
**Activation of ERK and p38 MAPK but not JNK signaling pathways by AGE-BSA**. Representative western blots of ERK, phosphorylated ERK, p38 MAPK, phosphorylated p38 MAPK, JNK, and phosphorylated JNK in cells treated with AGE-BSA for 2 (A) and 6 hours (B). Phosphorylation of ERK and p38 MAPK was activated dose-dependently but no such effect was seen for JNK.

In addition, to understand the effect of AGE-BSA on protein synthesis at the transcription level, Cx43 transcripts was measured using semi-quantitative RT-PCR, which showed a dose-dependent decrease of the transcripts in cells treated with AGE-BSA for 24 hours (relative expression levels, 500 μg/ml of AGE-BSA, 57.1 ± 8.9%; p < 0.05 compared to the control group; see Figure 8A). This effect was more obvious after treatment for 48 hours (500 μg/ml of AGE-BSA, 38.3 ± 5.5%; p < 0.05; see Figure 8B). Moreover, the decrement of Cx43 transcripts at exposure of 500 μg/ml of AGE-BSA followed a time-dependent manner, as early as 6 hours, a significant decrease was noticed (77.5 ± 5.1%; p < 0.05; see Figure 8C). The down-regulation of Cx43 transcripts by AGE-BSA (500 μg/ml) seen at 24 hours was significantly reversed in cells pre-treated with PD98059 or SB203580 (without PD98059 or SB203580, 65.1 ± 2.1%; with PD98059, 95.1 ± 5.3%; with SB203580, 98.8 ± 9.4%, all p < 0.05; see Figure 8D). No affections on the basal levels of Cx43 transcripts were seen after the addition of these inhibitors. This indicated that the AGE-BSA-induced reduction in Cx43 transcription was mediated by ERK and p38 MAPK. Phosphorylation of ERK and p38 MAPK was activated dose-dependently in cells treated with AGE-BSA for 2 hours (Figure 9A) and 6 hours (Figure 9B), while no such change was seen in JNK. Taken together, these data indicated that the down-regulation of Cx43 expression was through ERK and p38 MAPK signaling pathways.

## Discussion

This study demonstrates that, in cultured HAEC, i) Cx43 is the predominant gap junction component protein, unlike HUVEC; ii) AGE-BSA reduced Cx43 transcript expression in a time-dependent manner; and iii) AGE-BSA reduced Cx43 protein expression and attenuated gap-junction intercellular communication in a dose-dependent manner; Although a substantial portion of HAEC died when the cells were treated with high dose of AGE-BSA (500 μg/ml) for more than 24 hours, the down-regulating effects of AGE-BSA on Cx43 expression should not be mainly attributed to a general reduction in protein expression during cell dying, since the expression level of actin protein along the reduction of Cx43 was relatively stable (see Figure 4). In addition, inhibition of gap-junction communication activity may cause cell death, as seen in retinal capillary cells, in which attenuation of gap-junction communication with high glucose treatment disturbed normal transport of small molecules and calcium ions, leading to cell death [14,19]. The reduction of Cx43 transcripts by AGE-BSA was partially recovered by inhibitors of each of the ERK1 and p38 MAPK. Moreover, the activation of the MAPK cascades was further confirmed by the increase of phosphorylated ERK and p38 after a short time exposure to AGE-BSA indicating that activation of both the ERK and p38 MAPK cascades is involved in this process. Despite the down-regulation of Cx43 expression by AGE-BSA can be abrogated by the inhibitors and the reversed Cx43 mainly stayed at the cell borders, the gap-junction communication was impaired. These novel findings are complementary to our current knowledge regarding the effects of diabetes on endothelial gap junctions.

The results of the present study, in conjunction with those from other in vitro experiments investigating the effect of high glucose on endothelial gap junctions [13,14], indicate that in the circulation of diabetic individuals high glucose per se as well as the glycated albumin, which is generated in high glucose milieu, affects endothelial gap junction. Interestingly, both the actions of high glucose and the glycated albumin on endothelial gap junctions may share similar mechanisms. In cultured microvasular endothelial cells treated with high glucose, the Cx43 mRNA was reduced [13]. The same effect was observed for the AGE-BSA-induced down-regulation of Cx43 in this study [14].

Phosphorylation of the members of connexin, including Cx43, is well known to play a key role in regulation of gap-junction communication [20]. The effects of phosphorylation on the communication can be either enhancing or inhibiting. The phosphorylation status of Cx43 is believed to affect the oligomerization of Cx43 into connexons and delivery of integral connexins to justified locations [21-23]. Of the kinases involved in phosphorylation of Cx43, ERK and p38 MAPK were reported to attenuate the gap-junction communication [24]. These data mainly came from non-endothelial cells. On the other hand, activation of the ERK and p38 MAPK cascades was also reported to affect the expression level of Cx43 protein. In cultured smooth muscle cells isolated from human saphenous vein, inhibition of ERK and p38 MAPK by using siRNA specific to the kinases were shown to attenuate the enhanced expression of Cx43 induced by angiotensin II [24]. Considering in the present study that i) a close association between the AGE-BSA-induced reduction in the Cx43 mRNA level and the reduction of Cx43 protein is appeared; ii) the down-regulation of endothelial Cx43 transcript and protein by AGE-BSA is blocked by inhibitors of kinase (ERK and p38 MAPK) cascades; and iii) phosphorylations of ERK and p38 MAPK were activated dose-dependently, one may question whether in endothelial cells after exposure to AGE-BSA there is a link between activation of the MAPK and enhanced proteolysis of Cx43, as reported in lens epithelial cells that phosphorylation is a signal for Cx43 proteins to enter a proteasome-dependent degradation [25]. Moreover, activation of the ERK and p38 MAPK cascades in endothelial cells exposed to AGE-BSA results in regulation of Cx43 protein in a direction opposite to that seen for smooth muscle cells [24]. Recent findings indicated that AGE-HSA (human serum albumin) could promote endothelial progenitor cells (EPCs) senescence and apoptosis via ERK and p38 MAPK pathways, leading to the down-regulation of the number of EPCs [26,27]. This decreases the protective capacity of EPCs on atherosclerosis in diabetic patients and increases their risks for cardiovascular diseases [28]. Similarly, glucose, AGE, and methylglyoxal, the precursor of AGE, could enhance apoptosis of human cardiac myocytes and collagen deposition, an important event of diabetic cardiomyopathy, in rat cardiac fibroblasts accompanied by temporal activation of ERK1/2, p38 MAPK, and nuclear O-GlcAcylation [13,29,30]. Taken together, our findings in the present study suggested that reduced Cx43 transcription is the major mechanism underlying the down-regulation of Cx43 protein in HAEC treated with AGE-BSA. More studies are required to determine whether an increased turnover rate of Cx43 plays a significant role in this process. Although the down-regulation of Cx43 expression was abolished by inhibitors, these rescued Cx43 proteins may already lose their membrane-association and consequently be unable to assemble properly to exert their activity. It is also possible that gap junctions were induced to partially close during the blockage of those signaling pathways. These two phenomenons were already reported in cells under certain physical and chemical offenses [31,32].

Down-regulation of Cx43 gap junctions in endothelial cells by AGE-BSA has implications in the pathogenesis of diabetic vasculopathy, which, as mentioned before, starts with endothelial dysfunction [1]. Our recent findings indicated that in endothelial cells down-regulation of Cx43 per se activates the cells to a pathological status, in which pro-coagulatory molecules, such as plasminogen activation inhibitor 1 and von Willebrand factor, were up-regulated and the viability, angiogenic capacity, and proliferation of the cells were reduced [33]. These changes of endothelial cells due to insufficient expression of Cx43 may further aggravate the harmful effects of AGE-BSA on the vascular cells.

## Conclusions

This study provided the evidence that AGE-BSA down-regulated Cx43 expression in HAEC, mainly through reduced Cx43 transcription, and this process involved activation of ERK and p38 MAPK.

## Competing interests

The authors declare that they have no competing interests.

## Authors' contributions

WC-Y, LH-J, CH-J designed, carried out the experiments, analyzed data and drafted the manuscript. LY-C performed the gap junction communication assay, analyzed data and commented on the manuscript. WH-H performed the real-time PCR, analyzed data and commented on the manuscript. HT-C designed experiments, analyzed data and commented on the manuscript. YH-I supervised the project and corrected the final manuscript. All authors read and approved the final manuscript.

## Supplementary Material

Additional file 1 **Figure S1 -Comparison of the effects of AGE-BSA and BSA on expression of Cx43 proteins, as detected by Western blotting**. No effects on the expression levels of Cx43 protein by BSA were detected. Note that a dose-dependent reduction is only seen in cells treated with AGE-BSA (A) but not with dialyzed, long-term stored BSA (B). Cells were treated for 24 hours. Click here for file

Additional file 2 **Figure S2 -Comparison of different lysis buffers in extraction of Cx43 proteins from HAEC treated with a serious concentrations of AGE-BSA, as detected by Western blotting**. No differences on the expression levels of Cx43 protein were detected using both extraction buffers. Note that no difference in decreasing trends or patterns of Cx43 proteins extracted from cells using either NP40 (A) or SDS (B) buffers. Click here for file

Additional file 3 **Figure S3 -Comparison of anti-Cx43 antibodies of various sources in detecting Cx43 proteins from cells treated with a serious concentrations of AGE-BSA, as examined by Western blotting**. No differences on the expression levels of Cx43 proteins were detected using both anti-Cx43 antibodies. Note the same pattern of a dose-dependent reduction was seen using antibodies of different sources (antibody used in A, from Chemicon; in B from, BD Biosciences). Click here for file
