# Supplementary figures and images for: AGE-BSA down-regulates endothelial connexin43 gap junctions
Source: BMC Cell Biol. 2011 May 16;12:19. doi: 10.1186/1471-2121-12-19 (PMC3224147; doi:10.1186/1471-2121-12-19)

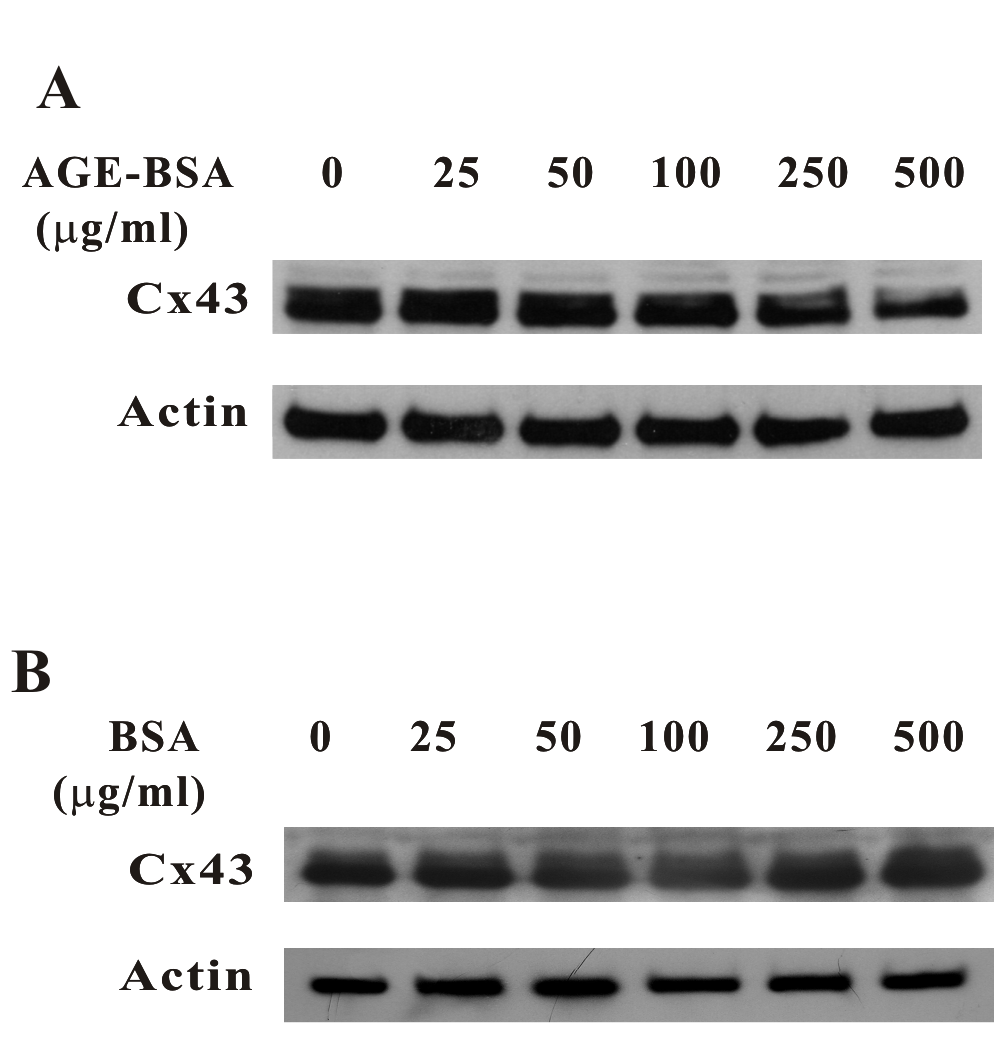

Supplement: Additional file 1 — Figure S1 -Comparison of the effects of AGE-BSA and BSA on expression of Cx43 proteins, as detected by Western blotting. No effects on the expression levels of Cx43 protein by BSA were detected. Note that a dose-dependent reduction is only seen in cells treated with AGE-BSA (A) but not with dialyzed, long-term stored BSA (B). Cells were treated for 24 hours. [file 1471-2121-12-19-S1.TIFF]

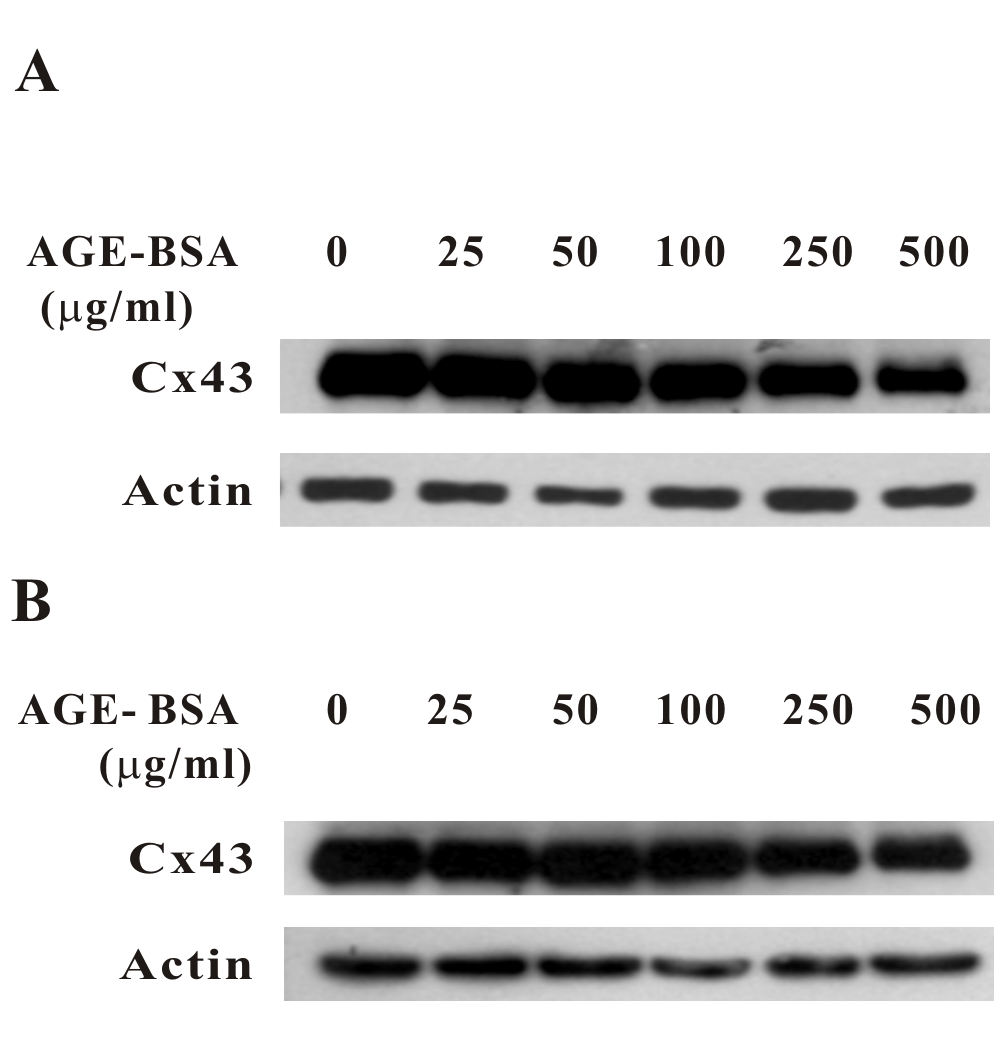

Supplement: Additional file 2 — Figure S2 -Comparison of different lysis buffers in extraction of Cx43 proteins from HAEC treated with a serious concentrations of AGE-BSA, as detected by Western blotting. No differences on the expression levels of Cx43 protein were detected using both extraction buffers. Note that no difference in decreasing trends or patterns of Cx43 proteins extracted from cells using either NP40 (A) or SDS (B) buffers. [file 1471-2121-12-19-S2.TIFF]

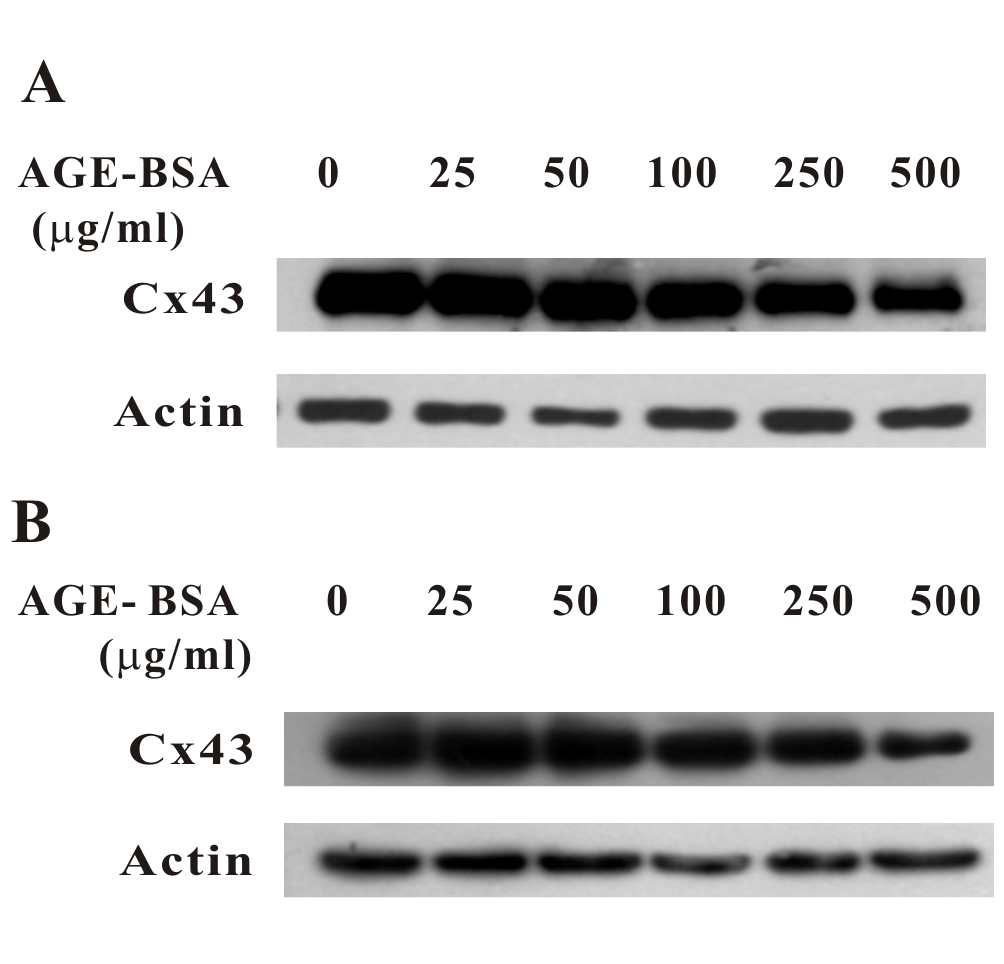

Supplement: Additional file 3 — Figure S3 -Comparison of anti-Cx43 antibodies of various sources in detecting Cx43 proteins from cells treated with a serious concentrations of AGE-BSA, as examined by Western blotting. No differences on the expression levels of Cx43 proteins were detected using both anti-Cx43 antibodies. Note the same pattern of a dose-dependent reduction was seen using antibodies of different sources (antibody used in A, from Chemicon; in B from, BD Biosciences). [file 1471-2121-12-19-S3.TIFF]
